# Supplementary material for: Extracellular Vesicles from Campylobacter jejuni CDT-Treated Caco-2 Cells Inhibit Proliferation of Tumour Intestinal Caco-2 Cells and Myeloid U937 Cells: Detailing the Global Cell Response for Potential Application in Anti-Tumour Strategies
Source: Int J Mol Sci. 2022 Dec 28;24(1):487. doi: 10.3390/ijms24010487 (PMC9820799; doi:10.3390/ijms24010487)
Supplement: Supplementary file 1 [file ijms-24-00487-s001.zip › ijms-2019020-supplementary.pdf]

SUPPLEMENTARY FIGURE

Extracellular vesicles from Campylobacter jejuni CDT-treated Caco-2 cells inhibit proliferation of tumour intestinal Caco-2 cells and myeloid U937 cells: detailing the global cell response for potential application in anti-tumour strategies

Mariele Montanari[1]; Michele Guescini[1], Ozan Gundogdu[2], Francesca Luchetti[1], Paola Lanuti [3,4], Caterina Ciacci[1], Sabrina Burattini[1], Raffaella Campana[1], Claudio Ortolani[1], Stefano Papa[1*] and Barbara Canonico[1]

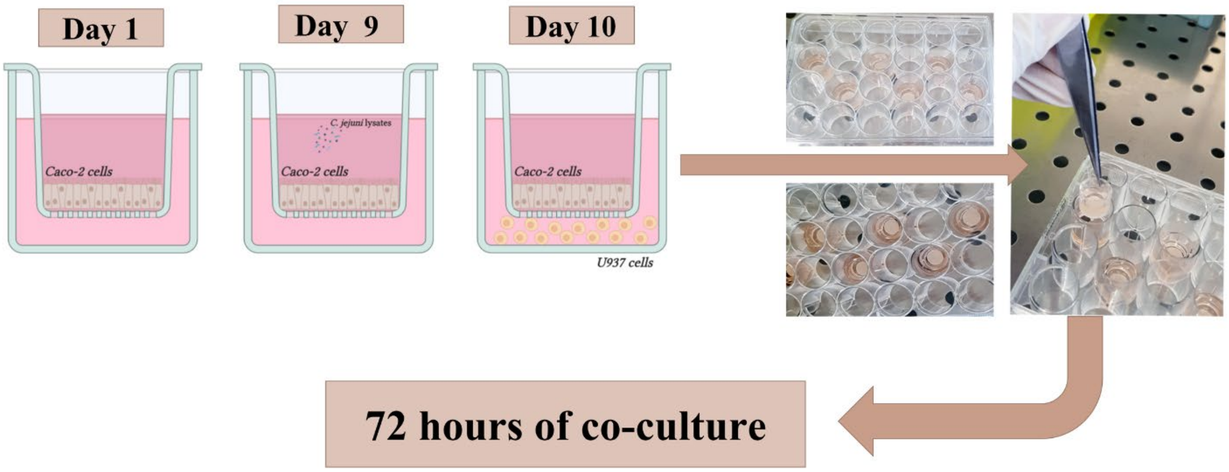

Figure S1. Schematic description of the co-culture set-up. The schematic diagram for the intestinal epithelial-myeloid cell co-culture (created in Biorender.com).

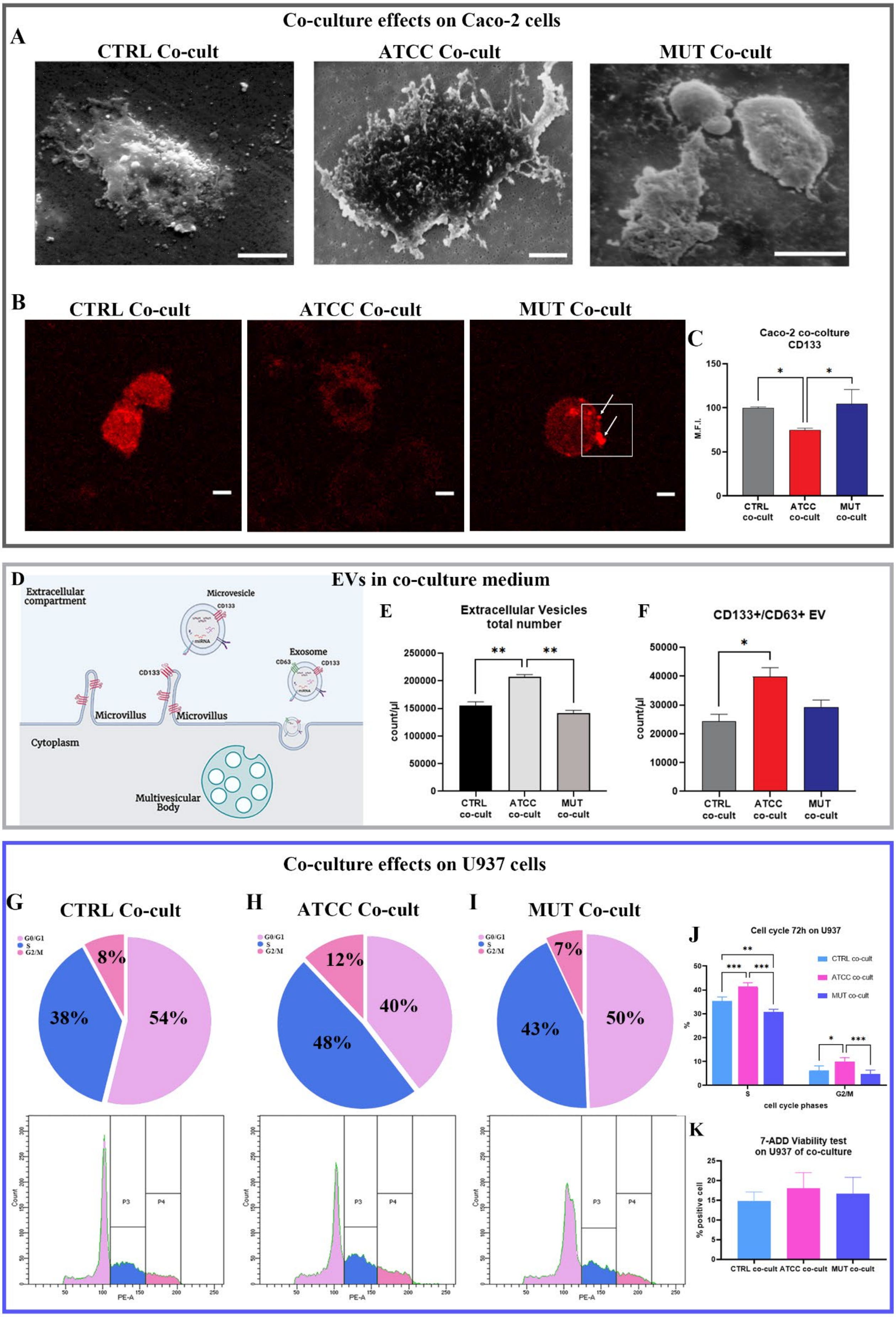

Co-culture effects on Caco-2 cells
A
CTRL Co-cult
ATCC Co-cult
MUT Co-cult
B
CTRL Co-cult
ATCC Co-cult
MUT Co-cult
C
Caco-2 co-colture CD133
M.F.I.
150
100
50
0
*
*
CTRL co-cult
ATCC co-cult
MUT co-cult
D
EVs in co-culture medium
Extracellular compartment
Microvesicle
CD133
miRNA
Exosome
CD63
CD133
Microvillus
Microvillus
Cytoplasm
Multivesicular Body
E
Extracellular Vesicles total number
count/µl
250000
200000
150000
100000
50000
0
**
**
CTRL co-cult
ATCC co-cult
MUT co-cult
F
CD133+/CD63+ EV
count/µl
50000
40000
30000
20000
10000
0
*
CTRL co-cult
ATCC co-cult
MUT co-cult
Co-culture effects on U937 cells
G
CTRL Co-cult
G0/G1
S
G2/M
8%
38%
54%
H
ATCC Co-cult
G0/G1
S
G2/M
12%
40%
48%
I
MUT Co-cult
G0/G1
S
G2/M
7%
43%
50%
P3
P4
Count
PE-A
J
Cell cycle 72h on U937
CTRL co-cult
ATCC co-cult
MUT co-cult
%
50
40
30
20
10
0
**
***
***
*
***
S
G2/M
cell cycle phases
K
7-ADD Viability test on U937 of co-culture
% positive cell
25
20
15
10
5
0
CTRL co-cult
ATCC co-cult
MUT co-cult

Figure S2. (**A**) SEM micrographs of Caco-2 co-culture for CTRL co-cult, ATCC co-cult and MUT co-cult. Bar represents 10 µm. (**B**) Single confocal optical sections of CD133 localization on detached Caco-2 cell at 72 h of co-culture. Bar represents 20µm (**C**) Statistical histograms of CD133 MFI value after 72 h of co-culture on Caco-2 cells. Each value is expressed as a mean ± SD. **(D)** Extracellular vesicles are cell secreted vesicles packed with a variety of cellular components including mRNAs, miRNAs, proteins, enzymes, lipids (Resuming Scheme). The EV surface is decorated with various membrane proteins responsible for different pathophysiological functions (created in Biorender.com). **(E)** Statistical histogram of Total EV-count from CTRL-co-culture, ATCC-co-culture, MUT-co-culture at 72 h. **(F)** Statistical histogram of absolute counting of Caco-2-released EV from CTRL-co-culture, ATCC-co-culture, MUT-co-culture at 72 h. Pie chart (above) and cytometric histograms (below) for cell cycle distribution of U937 CTRL-co-culture (**G**), ATCC-co-culture (**H**), MUT-co-culture (**I**). (**J**) Statistical histogram of S and G2/M phases of cell cycle calculated in cytometry via PI staining at 72 h. (**K**) Statistical histograms of the percentage of 7'AAD positive cells on U937 cells of CTRL-co-culture, ATCC-co-culture, MUT-co-culture. One or two-way ANOVA with Bonferroni's multiple comparison test. The asterisk denotes a statistically significant difference (* = $p < 0.05$, ** = $p < 0.01$, *** = $p < 0.001$).

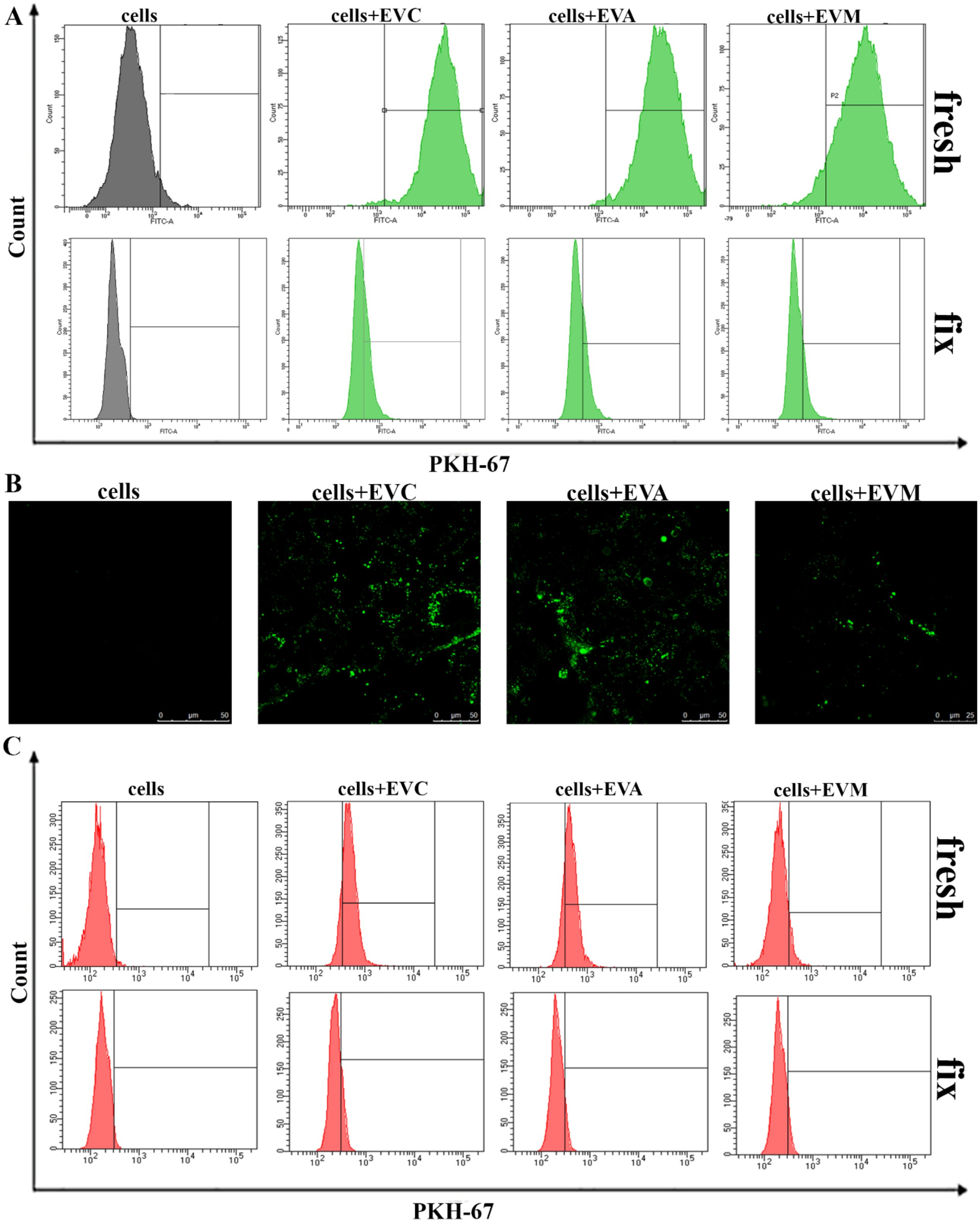

Figure S3. (A) Representative cytometric histograms of fresh (upper) and ethanol-fixed cells (below) for Caco-2 cells treated with EvC, EvA, EvM. (B) Single confocal optical sections of the Caco-2 cells treated with PKH67-labeled EvC, EvA and EvM at 48 h. Bar represents 20μm (C) Representative cytometric histograms of fresh (upper) and ethanol-fixed cells (below) for U937 cells treated with EvC, EvA, EvM.
